# Announcement: The Role of Ligands in Metallobiochemistry ACS National Symposium

**DOI:** 10.1155/MBD.1994.339

**Published:** 1994


					oL 1, No. 4, 1994 Announcements
THE ROLE OF LIGANDS IN
METALLOBIOCHEMISTRY
ACS NATIONAL SYMPOSIUM
Washington, D.C.
August 21 23, 1994
The following speakers meeting were invited to give a lecture during this meeting devoted to the role
of ligands in metallobiochemistry
Sunday, August 21 Tutorial
1:00 Larry Que
1:45 Brian Hoffman
2:30 Gerard Canters
3:15 William Woodruff
4:00 Michael Maroney
Monday, August 22,
i,m.
8:30 Gerard Canters
9:10 Joan Valentine
9:50 Les Dutton
10:30 Harry Gray
11:10 Stephen Cooper
11:50 Nick Farrell
p.m.
1:30 Stephen Lippard
2:10 Larry Que
2:50 Robert Buchanan
3:30 Jim Penner-Hahn
3:50 William Rutherford
4:30 David Britt
5:10 William Armstrong
Tuesday, August 23,
i,m.
8:30 Robert Huber
9:10 Brian Hoffman
9:50 William Woodruff
10:30 Ken Karlin
11:10 David Dooley
11:50 James Whittaker
1:30 Joanne Stubbe
2:10 Ed Stiefel
2:50 Steve Ragsdale
3:30 Mike Maroney
3:50 Marc Waiters
4:30 Louis Noodleman
5:10 Alan Balch
More information can be obtained from
William H. Armstrong
Department of Chemistry, Merkert Chemistry Center, Boston College
Chestnut Hill, MA 02167, USA
e-mail: Armstrong@Hermes.BC.edu
339

				

